# Treatment of Ethylene Glycol Poisoning with Oral Ethyl Alcohol

**DOI:** 10.1155/2019/7985917

**Published:** 2019-01-30

**Authors:** B. Achappa, D. Madi, T. Kanchan, N. K. Kishanlal

**Affiliations:** ^1^Associate Professor, Department of Internal Medicine, Kasturba Medical College, Manipal Academy of Higher Education, Mangalore, India; ^2^Associate Professor, Department of Forensic Medicine & Toxicology, All India Institute of Medical Sciences, Jodhpur, India; ^3^Senior Resident, Department of Internal Medicine, Kasturba Medical College, Manipal Academy of Higher Education, Mangalore, India

## Abstract

Ethylene glycol poisoning is not uncommon in India. The ill effects are primarily caused by its toxic metabolites: glycolic acid and oxalic acid. A 70-year-old female presented to our hospital with ataxia after ingestion of ethylene glycol. The reported case describes the management of ethylene glycol poisoning using oral ethyl alcohol as an alternative to the recommended intravenous ethyl alcohol and fomepizole that are not available for use in India. The need for high degree of clinical suspicion, targeted investigations, and early instigation of treatment is of prime importance in cases of ethylene glycol poisoning as it can lead to long-term complications or even death.

## 1. Introduction

Ethylene glycol popularly used as an antifreeze agent in the automobiles, is an odorless and sweet-tasting liquid, which makes it prone for accidental consumption. The ill effects are caused by its metabolism into toxic organic acids: glycolic acid and oxalic acid. Intravenous ethyl alcohol and fomepizole are the preferred drugs for the treatment of ethylene glycol poisoning [1]. Both these agents are competitive inhibitors of alcohol dehydrogenase, the enzyme responsible for metabolizing ethylene glycol into its toxic components. However, as the aforementioned drugs are not licensed for use in India, we administered oral ethyl alcohol as an alternative treatment for our patient who had ingested ethylene glycol accidentally. There are hardly any case reports from India where oral ethyl alcohol therapy was successfully used to treat ethylene glycol poisoning.

## 2. Case Report

A 70-year-old female was brought to the emergency department of our tertiary care referral hospital with an alleged history of accidental “car coolant” consumption followed by unsteadiness of gait about 3 hours later. On examination, the patient was drowsy but following verbal commands. There was no odour of alcohol in the breath. The GCS score was 15/15 (E4V5M6). Her blood pressure was 140/80 mm of Hg and pulse rate 68 per minute. Oxygen saturation was maintained at room air. ECG was normal, and no focal neurological deficit was detected. Gastric lavage was performed as the first line of treatment. Laboratory investigations revealed Na^+^ 142 mEq/L, K^+^ 2.4 mEq/L, Cl^−^ 101.1 mEq/L, HCO_3_^−^ 15.8 mEq/L, Ca^+^ 5 mg/dl, urea 35 mg/dl, creatinine 0.7 mg/dl, BUN 16.35 mg/dl, random glucose 141 mg/dl, and serum osmolality 323 mOsm/kg. Arterial blood gas analysis showed pH 7.322, pCO_2_ 30.7 mmHg, pO_2_ 93.8 mmHg, cBase(B)c −9.1. Urine examination revealed crystals of calcium oxalate. Anion gap was 25.1 mEq/L, and osmolar gap was 17 mOsm/kg·H_2_O. Blood and urine levels of ethylene glycol could not be obtained for lack of such analytical facilities in the region.

Crystalluria observed in the case is considered as a major indicator of ethylene glycol consumption [2]. Anion gap metabolic acidosis and high osmolar gap gave further confirmation to our diagnosis. Oral ethanol therapy was started at 2.5 ml/kg of 40% ethanol [3] through the nasogastric tube. In view of high anion gap metabolic acidosis, the patient was given hemodialysis (HD) over four hours with high potassium dialysate. Intravenous calcium gluconate was given over 10 minutes for management of hypocalcaemia. In addition, pyridoxine and thiamine were administered. She was given 100 ml of ethanol before dialysis.

Repeat arterial blood gas analysis was performed after 12 hours, which showed marked improvement in patient's condition. The patient improved clinically, and the investigations were in normal limits with pH 7.416, pCO_2_ 34.7 mmHg, pO_2_ 94.1 mmHg, and cBase(B)c −1.7. Calcium infusion was stopped. Repeat electrolytes were within normal limits with Na^+^ 141 mEq/L, K^+^ 5.2 mEq/L, Cl^−^ 101.3 mEq/L, HCO_3_^−^ 24.9 mEq/L, and Ca^+^ 9.4 mg/dl. Over the next 24 hours, she received 400 ml of ethanol through the nasogastric tube at timed intervals (approximately 35 ml second hourly).

Urine routine after 48 hours showed no crystalluria. The patient was clinically stable after 72 hours with laboratory parameters within normal range. No residual organ damages were detected upon follow-up.

## 3. Discussion

The toxicity of ethylene glycol can be described in three phases [4]: early toxicity (up to 12 hours) comprising a central nervous system depressant phase during which the patient develops stupor, vomiting, and seizures. Then, there is a cardiorespiratory phase (12 to 24 hours) after intoxication that appears with the onset of tachypnea and hypotension or congestive heart failure. Finally, 24 hours after ingestion, the patients develop flank pain and oxalate crystalluria, often followed by oliguria and acute kidney injury [5]. The toxic and lethal dose of 100% ethylene glycol is documented as approximately 0.2 ml/kg and 1.4 ml/kg, respectively [6]. The majority of ethylene glycol (nearly 80%) is broken down into glycolic acid and oxalic acid by alcohol dehydrogenase in the liver [7]. These metabolites are responsible for high anion gap metabolic acidosis, calcium oxalate formation, and further organ damage. Oxalic acid combines with calcium to form calcium oxalate, so hypocalcemia may occur. There is a time lag between ingestion and appearance of symptoms as the symptoms of poisoning are due to the metabolites and not the compound itself. The earlier the treatment is started, better is the expected outcome. In a case reported by Nagesh et al., a 48-year-old patient who reported to the hospital approximately 5 hours after consumption of ethylene glycol had already developed severe complications and later succumbed to it because of severe organ damages [7].

Intravenous ethanol, fomepizole, and hemodialysis are the most important therapies in patients with ethylene glycol poisoning [8, 9]. The American Academy of Clinical Toxicology recommends fomepizole as the preferred treatment in such cases. Fomepizole is a comparatively newer agent with specific indication for ethylene glycol poisoning and has been approved by the US Food and Drug Administration [1, 10, 11]. It has distinct advantage over others because it is easy to titrate and does not cause central nervous system depression, hypoglycemia, etc [12]. In a case report by Buchanan et al., a patient with high doses of ethylene glycol consumption was treated with fomepizole alone without hemodialysis (HD); they went on to suggest that treatment with fomepizole can be feasible in even high doses of ethylene glycol consumption, provided the renal function is maintained [13]. Similar case report was documented by Velez et al., where the patient with massive dose of ethylene glycol consumption was treated with fomepizole and basic supportive measures [14].

Both ethanol and fomepizole inhibit alcohol dehydrogenase in the initial step of the metabolic. Since fomepizole and IV ethyl alcohol are not available in India, we administered oral ethanol through the nasogastric tube to our patient. Welman et al. have used oral ethanol to treat ethylene glycol poisoning in the UK [15]. They were able to monitor blood levels of ethanol and ethylene glycol in their patient. Laher et al. [16] have used oral ethanol to treat three cases of ethylene glycol poisoning in Africa. Target ethanol concentration in such treatment is set at 100–150 mg/dl [1]. Maintaining adequate ethanol levels is difficult in everyday practice; therefore, frequent testing and infusion adjustments are mandatory. We could not monitor blood levels of ethanol in our case due to lack of lab facilities. We dialyzed our patient in view of high anion gap metabolic acidosis. Eventually, she improved with oral ethyl alcohol and HD. Acidosis was corrected, urine output resumed, and she was discharged with no residual complications. Treatment with hemodialysis is recommended in the setting of known ethylene glycol ingestion if either there is a high anion gap metabolic acidosis, regardless of drug level, or there is evidence of end-organ damage [4, 5]. Mortality in ethylene glycol poisoning is around 27% [17].

Ethylene glycol poisoning remains an underdiagnosed cause of metabolic acidosis, especially in the emergency departments. Detailed medical history is not always available due to the altered mental status of some patients at the time of presentation. In all cases with high anion gap metabolic acidosis and high osmolar gap, ethylene glycol poisoning must be considered [12, 16] unless there is any other definite cause suggested. Estimation of blood levels of ethylene glycol is not a readily available facility in the Indian scenario, so we need to rely more on strong index of suspicion, routine laboratory investigations, and clinical findings.

The reported case highlights on the importance of oral ethyl alcohol therapy where intravenous ethyl alcohol and fomepizole are not available. Oral ethanol when supplemented with timely hemodialysis (HD) leads to clinical improvement with no residual complications. The elimination half-life of ethylene glycol is approximately 3 hours [18]; hence, patients presenting to the hospital much later are not likely to be benefitted from ethanol therapy irrespective of the route of administration. In such cases, hemodialysis must be chosen as the main modality of treatment to remove the metabolites of ethylene glycol from the system. Our patient presented early to the emergency department with a definite history of coolant consumption, she had preserved renal function, and we started treatment at the earliest which led to a good outcome. Our case report will help physicians in managing a case of ethylene glycol poisoning especially in resource-limited settings.
